# Apigenin Induces Autophagy and Cell Death by Targeting EZH2 under Hypoxia Conditions in Gastric Cancer Cells

**DOI:** 10.3390/ijms222413455

**Published:** 2021-12-15

**Authors:** Tae Woo Kim, Hee Gu Lee

**Affiliations:** 1 Immunotherapy Research Center, Korea Research Institute of Bioscience and Biotechnology, Daejeon 34141, Korea; 2 Department of Preventive Medicine, College of Korean Medicine, Kyung Hee University, 1 Hoegi, Seoul 130-701, Korea; 3 Department of Biomedicine & Health Sciences, College of Medicine, The Catholic University of Korea, Seoul 06591, Korea; 4 Department of Biomolecular Science, University of Science and Technology, Daejeon 34113, Korea

**Keywords:** apigenin, autophagy, ER stress, hypoxia, resistance

## Abstract

Hypoxia is a major obstacle to gastric cancer (GC) therapy and leads to chemoresistance as GC cells are frequently exposed to the hypoxia environment. Apigenin, a flavonoid found in traditional medicine, fruits, and vegetables and an HDAC inhibitor, is a powerful anti-cancer agent against various cancer cell lines. However, detailed mechanisms involved in the treatment of GC using APG are not fully understood. In this study, we investigated the biological activity of and molecular mechanisms involved in APG-mediated treatment of GC under hypoxia. APG promoted autophagic cell death by increasing ATG5, LC3-II, and phosphorylation of AMPK and ULK1 and down-regulating p-mTOR and p62 in GC. Furthermore, our results show that APG induces autophagic cell death via the activation of the PERK signaling, indicating an endoplasmic reticulum (ER) stress response. The inhibition of ER stress suppressed APG-induced autophagy and conferred prolonged cell survival, indicating autophagic cell death. We further show that APG induces ER stress- and autophagy-related cell death through the inhibition of HIF-1α and Ezh2 under normoxia and hypoxia. Taken together, our findings indicate that APG activates autophagic cell death by inhibiting HIF-1α and Ezh2 under hypoxia conditions in GC cells.

## 1. Introduction

Natural compounds extracted from many plants, including fruits, vegetables, and traditional medicines, have properties of high bioavailability, low side effects, and potent pharmacological activity [1]. These natural products are often readily available in their many different structures, meaning that a novel compound can be developed from them [2]. There are many types of phytochemicals, including alkaloids, carotenoids, N-rich compounds, organo-sulfur compounds, and phenolics [3]. Furthermore, phenolics are known as the most abundant antioxidants and are divided into five types: phenolic acids, stilbenes, flavonoids, tannins, and coumarins [4]. Flavonoids are being increasingly investigated for their powerful anti-cancer effects against apoptosis, cell death, proliferation, inflammation, angiogenesis, metastasis, and chemoresistance [5]. According to recent studies on flavonoids, a compound known as hesperetin exerts anti-cancer effects by inducing apoptotic cell death via inhibition of Bax and Bcl-2 and ROS production [6]. Naringenin is found abundantly in fruits such as grape and orange and exerts anti-cancer effects via intrinsic and extrinsic apoptosis pathways in various cancer types [7]. Quercetin induces pro-apoptotic potential by suppressing the PI3K/Akt/mTOR and Stat3 pathways and mediates the p53 pathway in various cancer types, including leukemia, breast cancer, lymphoma, pancreatic cancer, and liver cancer [8,9,10,11]. Kaempferol reportedly induces cell death via ER stress in HepG2 cells and induces autophagic cell death via ER stress in gastric cancer cells [12,13]. APG is highly concentrated in vine spinach, orange, garlic, parsley, celery, carrot, propolis, artichokes, oregano, and chamomile and is a well-known anti-cancer agent [14]. Recent studies reported that APG has anti-cancer efficacy through epigenetic modification, ROS generation, and DNA damage and by inhibiting proliferation, angiogenesis, and inflammation in diverse cancers [15,16]. APG affects well-known pathways, including IGF, NF-κB, Stat3, p53, DNA damage, and cell cycle arrest pathways, since various signaling pathways regulate anti-cancer properties [17]. In particular, though APG induces apoptosis and cell death via NF-κB, ROS, and inflammation in gastric cancer, deep and detailed mechanisms underlying these activities remain unclear.

The ER is a principal intracellular organelle and plays diverse functions, such as protein folding, synthesis, transport, and cellular homeostasis maintenance [18]. ER stress induces cell death via activation of the unfolded protein response (UPR) in the tumor environment and overcomes chemoresistance against tumor therapy [19]. Various intracellular stresses, such as nutrient deprivation, hypoxia, and extracellular stress, inhibit tumor growth, and accumulated stresses induce cell death via the activation of ER stress and the UPR [20]. The three ER transmembrane proteins, namely, (PKR)-like endoplasmic reticulum kinase (PERK), inositol-requiring protein 1α (IRE1α), and activating transcription factor 6 (ATF6), were considered transducers to initiate the UPR pathway, and the binding of PERK, IRE1α, or ATF6 with glucose-regulated protein 78 (GRP78) dissociates when ER stress is induced [21]. A recent report suggested that many therapeutic flavonoids mediate cell death via prolonged ER stress in cancer cells [22]. Chrysin, a natural flavonoid, induces mitochondria-mediated cell death via the loss of mitochondrial membrane potential (MMP) and ROS generation and ER stress-mediated cell death via the PERK–eIF2α–ATF4–CHOP signaling in prostate cancer cells [23]. Wogonin is a potential anti-cancer agent and flavonoid for gastric cancer because it blocks tumor growth and expansion and causes apoptotic cell death through ROS and ER stress, indicating phosphorylation of PERK and eIF2α [24]. Accumulating reports have indicated that autophagy was frequently regulated by the ER stress response, and the relationship between ER stress and autophagy is an important signaling pathway for anti-cancer studies [25]. With the ER stress function, autophagy, which means “self-eating,” also regulates cell homeostasis and is activated via diverse stress stimuli, including hypoxia, amino acid starvation, and nutrient deprivation [26]. The ER stress sensors PERK and IRE1α mediate autophagosome formation via up-regulation of LC3-II and puncta and consequently cause cell death [27]. In PERK signaling, ATF4 plays a functional role in the transcription of autophagy-related genes (e.g., LC3B, ATG5, AMP activated protein kinase α (AMPKα), and ULK1) since ER stress regulates the autophagy process [28]. In the autophagy process, mTOR is known as a negative regulator, whereas AMPKα directly activates autophagy [29]. mTOR regulates ribosomal protein S6 kinase 1 (S6K1; p70S6K) and eukaryotic initiation factor 4E (eIF4E)-binding protein 1 (4EBP1), and their activation and phosphorylation by mTOR promote protein synthesis and tumor growth [30]. Kaempferol causes cell death via the ER stress–autophagy axis in hepatocellular carcinoma cells, but the inhibition of CHOP blocks the autophagy pathway [31]. Under hypoxia, ER stress and the autophagy process regulate homeostasis, and ATF4 binds to LC3B and the CHOP promoter during PERK–eIF2α signaling and regulates autophagic flux and ER stress-induced cell death [32,33]. Hypoxia-caused stresses induce chemoresistance and cancer survival through the activation of hypoxia-inducible factor 1α (HIF-1α), pro-survival ER stress, and protective autophagy and consequently perturb cancer therapy in the tumor microenvironment [34]. Targeting HIF-1α could be a potential therapeutic strategy for overcoming resistance in cancer therapy, and a non-toxic flavonoid, namely, APG, frequently inhibits the activity of HIF-1α and has an anti-tumor effect in hypoxic environments [35,36,37,38]. Recently, it was found that mTOR signaling contributes to the accumulation of HIF-1α expression in solid tumors, and that hypoxia mediates the increased synthesis of the HIF-1α protein when compared to normoxia conditions [39]. Solid tumors in the hypoxia microenvironment are often regulated by HIF-1α, a master regulator of metastasis, and HIF-1α regulates tumor proliferation, survival, invasion, and angiogenesis [40]. Silibinin, a safe and non-toxic flavonoid, inhibits the up-regulation of HIF-1α by regulating the suppression of mammalian target of rapamycin (mTOR), ribosomal protein S6 kinase (p70S6K), and eukaryotic initiation factor 4E-binding protein-1 (4E-BP1) [41]. Previous reports have suggested that mTOR activation regulates the inhibition of the AMPK-ULK1-LC3B pathway, but the activation of AMPK signaling initiates the autophagy process via the inhibition of the mTOR pathway [42]. However, the underlying mechanism concerning whether APG regulates HIF-1α and Ezh2 via the mTOR pathway under hypoxia in GC remains unknown.

In the present study, we sought to examine whether APG mediates autophagic cell death via ER stress in GC and whether APG inhibits the accumulation of HIF-1α via the mTOR pathway. We identified that APG causes autophagic cell death via the PERK–ATF4–CHOP axis and suppresses HIF-1α and Ezh2 by inhibiting mTOR signaling in GC. Thus, APG is a powerful GC tumor therapeutic strategy of targeting hypoxia/HIF-1α.

## 2. Results

### 2.1. APG Induces Apoptotic Cell Death in GC Cells

**Figure 1:**
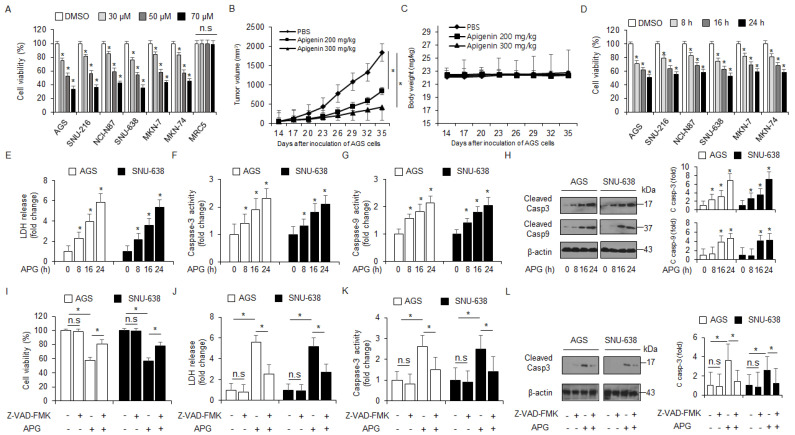
Cytotoxic effects of APG in GC cell lines. (**A**,**D**) Cell viability of dose- (0, 30, 50, and 70 µM, 24 h) and time-dependent (50 µM; 8, 16, and 24 h) APG, in GC cell lines, including AGS, SNU-216, NCI-N87, SNU-638, MKN-7, and MKN-74, and lung normal cell line MRC5 measured using the WST-1 assay on 96-well plates. (**B**,**C**) AGS cells (1 × 10^7^) were implanted (sc) into the thigh on the right hind leg of nude mice (*n* = 10/group). APG (200 or 300 mg/kg) or PBS was administered intraperitoneally (ip) once a day for two days. The longest (L) and shortest (W) axes of the tumors were measured, and the tumor volume (mm^3^) was calculated as LW2/2. Body weights of the AGS tumor xenograft mice were determined twice a week during the experiment. (**E**–**H**) APG was treated in a time-dependent manner (50 µM; 8, 16, and 24 h), and the LDH assay and caspase-3 and caspase-9 activity assays were performed. Western blot analyses of cleaved caspase-3 and -9 for the indicated times in APG-treated AGS and SNU-638 cells; * *p* < 0.05. β-actin was used as a protein loading control. (**I**–**L**) The effect of Z-VAD-FMK (50 μM) and APG treatment. AGS and SNU-638 cells were pre-treated with Z-VAD-FMK for 4 h and were subsequently treated with APG (50 μM, 24 h). Cell viability was determined using the WST-1 assay; cell cytotoxicity was monitored using the LDH assay, and caspase-3 activity was assessed using the caspase-3 activity assay; * *p* < 0.05, n.s; no signaificant. Sampling of total lysates was performed using the Western blot assay to identify the activation of apoptosis marker cleaved caspase-3. β-actin was used as a protein loading control.

To examine the cytotoxicity of APG in GC, we assessed the effect of APG on the viability of cells in a dose- and time-dependent manner (Figure 1A,D). APG mediated a dramatic reduction in cell viability in various GC cell types when compared to the control, but there was no change in MRC5 (Figure 1A). To validate the effects of APG in vivo, a gastric cancer xenograft mouse model was constructed using AGS cells. Mice in the 200 mg/kg and 300 mg/kg APG groups exhibited lower tumor volumes than those in the control group (Figure 1B). The body weights of all groups were not significant (Figure 1C). Next, we examined the LDH release and caspase-3 and caspase-9 activities of APG-treated AGS and SNU-638 cells in a time-dependent manner; consequently, LDH release and caspase-3 and caspase-9 activities were found to be increased time-dependently after APG treatment (Figure 1E–G). In Western blot analyses, time course experiments indicated that APG increased caspase-3 and caspase-9 cleavage in AGS and SNU-638 cells (Figure 1H). This finding suggests that APG exerts an anti-cancer effect via apoptosis and cell death in GC cells. Additionally, AGS and SNU-638 cells were treated with APG (30 µM) + Z-VAD-FMK (a pan-caspase inhibitor, 50 µM). Z-VAD-FMK + APG sufficiently suppressed the reduction in cell viability, increase in LDH release, and caspase-3 activity (Figure 1I–K). In Western blot analyses, Z-VAD-FMK + APG also inhibited caspase-3 cleavage to a greater extent than APG alone (Figure 1L). Our result shows that APG leads to an improved anti-cancer effect and mediates caspase-dependent cell death in GC cells.

### 2.2. APG Induces Autophagic Cell Death in GC Cells

**Figure 2:**
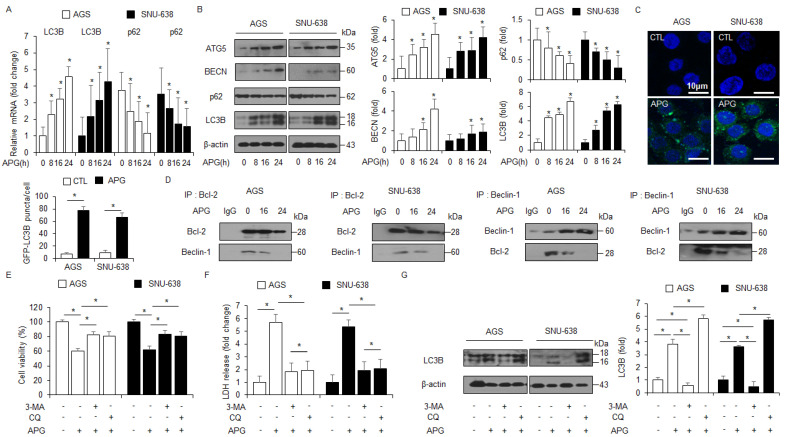
Stimulation of autophagy in APG-mediated GC cell lines. (**A**) Real-time RT-PCR analyses of LC3B and p62 in AGS and SNU-638 cells treated with APG (50 μM) for the indicated times. (**B**) Western blot analyses of ATG5, Beclin-1, p62, and LC3B protein levels in AGS and SNU-638 cells treated with APG (50 μM) for the indicated times. (**C**) AGS and SNU-638 cells treated with or without APG (50 μM) for 8 h were stained using the Cyto-ID autophagosome detection kit and analyzed as described in the Materials and Methods section. Fluorescence microscopy analysis analyzed by puncta of autophagosome staining; * *p* < 0.05. (**D**) AGS and SNU-638 cells were treated with APG (50 μM) for the indicated times. Bcl-2 was immunoprecipitated in AGS and SNU-638 cells, and the immunoprecipitated proteins were subjected to Western blot analyses. Beclin-1 was detected in immunoprecipitates prepared with anti-Bcl-2 antibody by immunoprecipitation. Bcl-2 was also monitored in immunoprecipitates prepared with anti-Beclin-1 antibody by immumoprecipitation. (**E**–**G**) Cell viability and LDH release were analyzed using WST-1 and LDH cytotoxicity assays in APG-treated AGS and SNU-638 cells with 3-MA (5 mM) or CQ (20 μM) treatment; * *p* < 0.05. Western blot analyses of LC3B in APG-induced AGS and SNU-638 cells with 3-MA (5 mM) or CQ (20 μM). β-actin was used as a protein loading control.

Real-time RT-PCR and Western blot analyses were carried out to identify the autophagy markers, including ATG5, Beclin-1 (BECN), p62, and LC3B, in a time-dependent manner. APG treatment up-regulated ATG-5, Beclin-1, and LC3-II and decreased p62 levels to a greater extent than the control treatment (Figure 2A,B). To determine whether APG mediates the autophagy process and autophagosome formation in GC cells, the autophagosome detection assay was performed to identify autophagosome formation. When AGS and SNU-638 cells were treated with APG, we analyzed puncta and counted approximately 4~5-fold more puncta with APG when compared to the control (Figure 2C). To identify the interaction of Beclin-1–Bcl-2 complex in APG-mediated GC cells, co-immunoprecipitation (IP) using antibodies for Beclin-1 and Bcl-2 was confirmed (Figure 2D). Our finding shows that the interaction between Beclin-1 and Bcl-2 was lowered in APG-mediated cells compared to control cells. To confirm the biological role of APG in the regulation of autophagic flux in AGS and SNU-638 cells, 3-methyladenine (3-MA, an inhibitor of the PI3 complex and autophagosomes) and chloroquine (CQ, an inhibitor of autophagosomes/lysosomes) were co-treated with APG, and cell viability and LDH assays along with Western blot analysis were performed. 3-MA/APG or CQ/APG treatment inhibited the reduction in cell viability and the increase in LDH release to a greater extent than APG treatment alone (Figure 2E,F). In Western blot analyses, 3-MA/APG inhibited the expression of LC3-II, indicating the suppression of autophagosome formation, and CQ/APG induced the accumulation of LC3-II, indicating the inhibition of the fusion of autophagosomes with lysosomes (Figure 2G). These results suggest that APG regulates autophagic flux in GC cells.

### 2.3. Autophagy Inhibition Blocks APG-Induced Autophagic Cell Death in GC Cells

**Figure 3:**
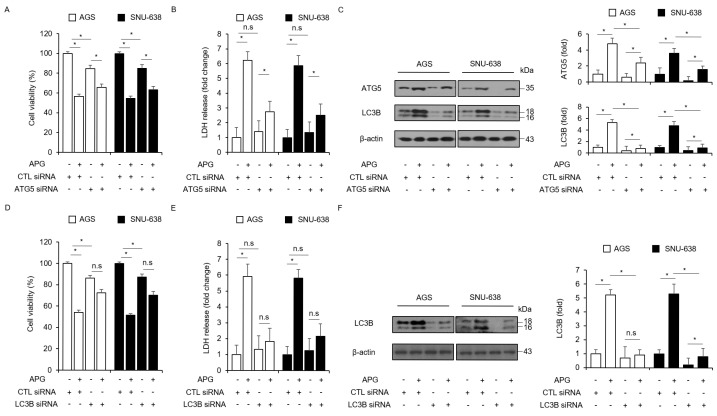
Autophagy inhibition regulates APG-induced cell death. (**A**–**F**) After AGS and SNU-638 cells were transfected with LC3B and ATG5 siRNAs, cell viability, LDH production, and Western blot analyses were performed with/without APG (50 μM, 24 h) treatment. Cell viability and LDH activity were determined using WST-1 and LDH assays, respectively; * *p* < 0.05, n.s; no significant. Western blotting was performed to identify the autophagy-related genes ATG5 and LC3B in APG-treated ATG5 or LC3B knockdown cells. β-actin was used as a protein loading control.

To further investigate if APG regulates autophagic cell death, LC3B- and ATG5-specific siRNAs were transfected into AGS and SNU-638 cells and then treated with APG. Transfection of LC3B and ATG5 siRNAs indicated better cell viability and lower LDH release than transfection of control siRNAs (Figure 3A,B,D,E). Compared to controls, the transfection experiment involving LC3B and ATG5 knockdown reduced LC3B and ATG5 levels in APG-mediated AGS and SNU-638 cells to a greater extent (Figure 3C,F). These results indicate that targeting autophagy blocks autophagic cell death by APG treatment in GC cells.

### 2.4. APG Induces Autophagic Cell Death via mTOR-AMPK-ULK1 Pathway in GC Cells

**Figure 4:**
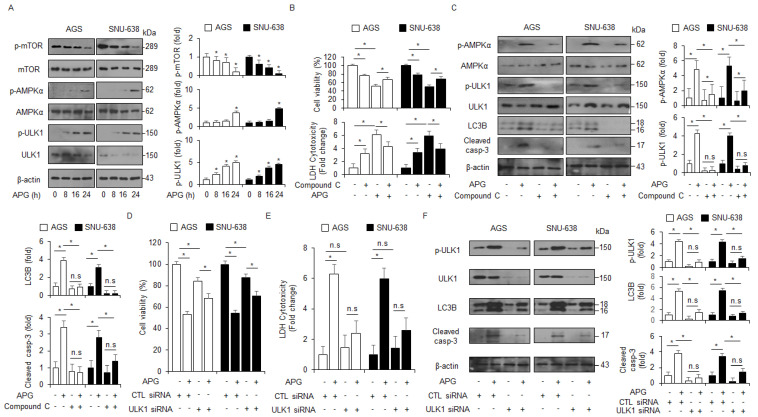
AMPKα–ULK1 axis modulates autophagic cell death in APG-treated GC cells. (**A**) AGS and SNU-638 cells were treated with APG (50 μM) in a time-dependent manner (0, 8, 16, and 24 h). Western blot analyses were performed for p-mTOR, mTOR, p-AMPKα (Thr172), AMPKα, p-ULK1 (Ser555), ULK1, p-mTOR (Ser2448), and mTOR in APG-treated AGS and SNU-638 cells. β-actin was used as a protein loading control. (**B**,**C**) Cell viability, LDH release, and Western blot analyses were performed for p-AMPKα, AMPKα, p-ULK1, ULK1, cleaved caspase-3, and LC3B in APG (50 μM, 24 h)-induced AGS and SNU-638 cells in the presence or absence of compound C (2 μM, 24 h); * *p* < 0.05, n.s; no significant. β-actin was used as a protein loading control. (**D**–**F**) Cell viability, LDH release, and Western blot analyses of p-ULK1, ULK1, LC3B, and cleaved caspase-3 in AGS and SNU-638 cells treated with APG (50 μM, 24 h) in the presence or absence of ULK1 siRNA (30 nM, 24 h); * *p* < 0.05, n.s; no significant. β-actin was used as a protein loading control.

Under autophagy activation, AMPK mediates ULK1 and interacts with ULK1 by inhibiting mTOR activity [43]. In an APG-treated time-dependent manner, Western blot analyses were performed to assess whether the mTOR/AMPKα signaling was regulated by APG treatment in GC cells. APG decreased the phosphorylation of mTOR and increased the activation of AMPKα and ULK1 (Figure 4A). In pharmacological experiments, compound C (AMPK inhibitor, 2 µM) in combination with APG increased cell viability and reduced LDH release to a greater extent than APG alone (Figure 4B). Compound C + APG caused the down-regulation of p-AMPKα, p-ULK1, LC3 lipidation, and caspase-3 cleavage in GC cells to a greater extent than APG alone (Figure 4C). To further investigate whether APG induces autophagic cell death, GC cells were transfected with ULK1-specific siRNA and then treated with APG. This result indicates that ULK1 knockdown increased cell viability and decreased LDH release in APG-treated GC cells to a greater extent than the control (Figure 4D,E). In Western blot analyses, ULK1 knockdown/APG resulted in the reduction in p-ULK1, LC3 lipidation, and caspase-3 cleavage in GC cells to a greater extent when compared to the control (Figure 4F). Therefore, these findings show that APG mediates autophagic cell death by regulating mTOR-AMPK-ULK1 signaling in GC.

### 2.5. APG Induces Autophagic Cell Death via ER Stress in GC Cells

**Figure 5:**
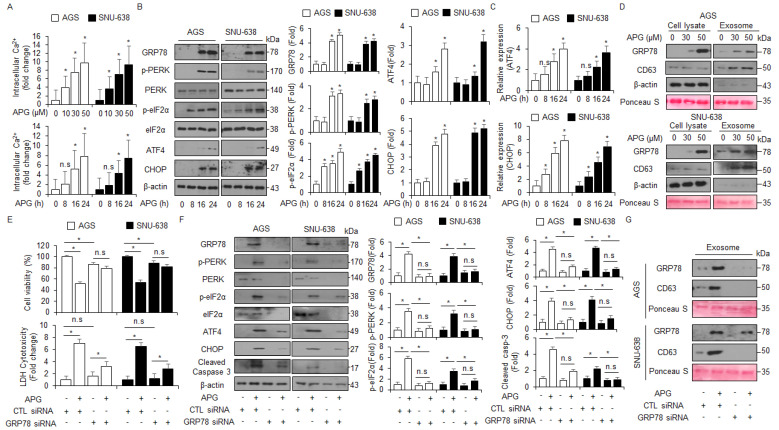
APG induces ER stress and cell death via intracellular Ca^2+^ release. (**A**) AGS and SNU-638 cells were treated with APG (50 μM) in a dose- (0, 10, 30, and 50 μM; 24 h) and time-dependent manner (0, 8, 16, and 24 h; 50 μM), and intracellular Ca^2+^ release was determined using intracellular Ca^2+^ assay. (**B**) AGS and SNU-638 cells were treated with APG (50 μM) for the indicated times, and the activation of ER stress signaling, including GRP78, p-PERK, PERK, p-eIF2α, eIF2α, ATF4, and CHOP, was assessed using the Western blot assay. β-actin was used as a protein loading control. (**C**) AGS and SNU-638 cells were treated with APG (50 μM) in a time-dependent manner, and mRNA levels of ATF4 and CHOP were investigated using real-time RT-PCR. (**D**) AGS and SNU-638 cells were treated with APG at the indicated doses (0, 30, and 50 μM, 24 h), and exosomes were extracted from the cell culture media. Protein samples extracted from cell lysates and exosomes were quantified by Ponceau S staining. These samples were obtained by Western blotting using the ER stress marker GRP78 and the exosome marker CD63. (**E**) AGS and SNU-638 cells were transfected with GRP78-specific siRNA in the presence or absence of APG (50 μM, 24 h), and cell viability and LDH assays were performed; * *p* < 0.05, n.s; no significant. (**F**) Western blot analysis of GRP78, p-PERK, PERK, p-eIF2α, eIF2α, ATF4, CHOP, and cleaved caspase-3 in APG (50 μM, 24 h)-treated AGS and SNU-638 cells in the presence or absence of GRP78 siRNA (30 nM, 24 h). β-actin was used as a protein loading control. (**G**) Western blot analysis of GRP78 and CD63 in exosomes extracted from APG (50 μM, 24 h)-treated AGS and SNU-638 cell culture media in the presence or absence of GRP78 siRNA (30 nM, 24 h). β-actin and Ponceau S were used as protein loading controls.

Emerging reports suggest that the crosstalk between ER stress-mediated cell death and autophagic cell death plays an important role in exerting an anti-cancer effect in tumor therapy, and the discovery of a signaling pathway connecting ER stress and autophagy will be required to study this mechanism [44]. To confirm intracellular calcium (Ca^2+^) release in APG-treated GC cells, an intracellular calcium (Ca^2+^) assay was performed, and we found that APG exerts intracellular Ca^2+^ release in a dose- and time-dependent manner (Figure 5A). To identify whether APG causes ER stress-medicated cell death in GC cells, we investigated ER stress-related proteins such as GRP78, p-PERK, PERK, p-eIF2α, eIF2α, ATF4, and CHOP. In Western blot analyses, APG increased GRP78, p-PERK, p-eIF2α, ATF4, and CHOP in a time-dependent manner (Figure 5B). In real-time RT-PCR, APG also increased ATF4 and CHOP in a time-dependent manner (Figure 5C). A robust report recently found that the ER stress marker GRP78 is released and secreted from various cancer cell types [45]. To confirm the induction of GRP78, the exosomes were isolated from APG-treated GC cell-cultured media, and Western blot analyses were performed. APG treatment increased GRP78 and exosome marker CD63 levels in a dose-dependent manner (Figure 5D). To confirm whether APG induces exosomal GRP78 from supernatant media, the cells were treated with APG after transfection with GRP78-specific siRNA, and WST-1 and LDH assays were performed along with Western blot analysis. Consequently, APG increased cell viability and reduced LDH release to a greater extent in GRP78 knockdown GC cells than the controls (Figure 5E). In Western blot analyses using cell lysate samples, APG decreased GRP78, p-PERK, PERK, p-eIF2α, eIF2α, ATF4, and CHOP in GRP78 knockdown GC cells to a greater extent when compared to controls (Figure 5F). Moreover, in Western blot analyses using exosome samples, APG decreased GRP78 and CD63 in GRP78 knockdown GC cells to a greater extent when compared to controls (Figure 5G). These results show that APG mediates the up-regulation of GRP78 through exosomes, and that this effect causes ER stress-induced cell death in APG-treated GC cells.

### 2.6. PERK Inhibition Blocks APG-Mediated Autophagic Cell Death in GC Cells

**Figure 6:**
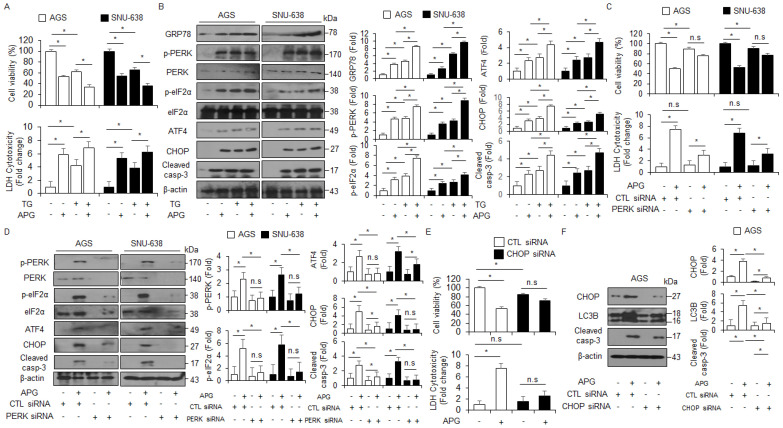
Targeting of ER stress signaling regulates APG-induced autophagic cell death in GC cells. (**A**,**B**) Western blotting of GRP78, p-PERK, PERK, p-eIF2α, eIF2α, ATF4, CHOP, and cleaved caspase-3 levels was determined using WST-1 and LDH assays in thapsigargin (3 μM, 24 h)- and APG (50 μM, 24 h)-treated GC cells; * *p* < 0.05. (**C**–**F**) After AGS and SNU-638 cells were transfected with PERK (30 nM, 24 h) and CHOP (30 nM, 24 h) siRNAs, cell viability, LDH production, and Western blot analyses were performed with/without APG (50 μM, 24 h) treatment. Cell viability and LDH activity were determined using WST-1 and LDH assays, respectively; * *p* < 0.05, n.s; no significant. Western blotting was performed to identify the ER stress-related genes p-PERK, PERK, p-eIF2α, eIF2α, ATF4, CHOP, and caspase-3 cleavage in APG-treated PERK or CHOP knockdown cells. β-actin was used as a protein loading control.

To further identify whether APG regulates autophagic cell death via ER stress in GC cells, thapsigargin (ER stress inducer, TG, 3 µM) was co-treated with APG (30 µM) in GC cells, and WST-1 and LDH assays were performed along with Western blot analysis. TG + APG decreased cell viability and enhanced LDH release to a greater extent than TG or APG alone (Figure 6A). In Western blot analyses, TG + APG increased GRP78, p-PERK, p-eIF2α, ATF4, CHOP, and caspase-3 cleavage to a greater extent than TG or APG alone (Figure 6B). ER stress is a powerful inducer of autophagy, and the PERK–eIF2α axis enhances the autophagy process, autophagosome formation, and LC3-II conversion [46]. To confirm whether APG regulates autophagic cell death via PERK signaling, AGS and SNU-638 cells were transfected with PERK-specific siRNA and then treated with APG. Under this experimental condition, WST-1 and LDH assays were performed along with Western blot analysis. In PERK knockdown GC cells, APG enhanced cell viability and decreased LDH release to a greater extent than the control (Figure 6C). In Western blot analyses, APG decreased p-PERK, PERK, p-eIF2α, ATF4, CHOP, and caspase-3 cleavage in PERK knockdown GC cells to a greater extent than the control (Figure 6D). To further explore whether APG mediates autophagic cell death via ER stress, AGS and SNU-638 cells were transfected with CHOP-specific siRNA and then treated with APG. Additionally, WST-1 and LDH assays were performed along with Western blot analysis. In CHOP knockdown GC cells, APG enhanced cell viability and decreased LDH release to a greater extent than the control (Figure 6E). In Western blot analyses, APG decreased CHOP and LC3 lipidation and caspase-3 cleavage in CHOP knockdown GC cells to a greater extent than the control (Figure 6F). This finding indicates that APG mediates autophagic cell death via PERK signaling in GC cells.

### 2.7. APG Inhibits HIF-1α and Induces Cell Death under Hypoxia in GC Cells

**Figure 7:**
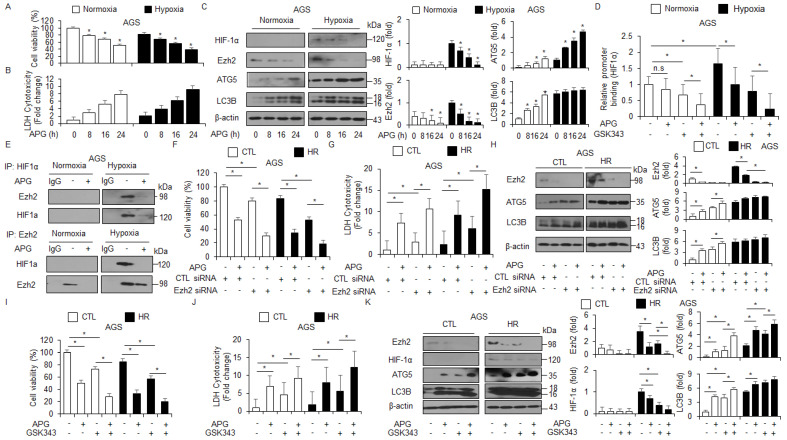
APG induces autophagic cell death via the inhibition of EZH2 in hypoxia-resistant GC cells. (**A**–**C**) AGS and AGS hypoxia-resistant (HR) cells with APG (50 μM) in a time-dependent manner. Cell cultures were also exposed to hypoxia or normoxia in a time-dependent manner. Cell viability and LDH release analyses were performed using WST-1 and LDH cytotoxicity assays; * *p* < 0.05. Western blot analysis was used to validate the targeted changes in HIF-1α, EZH2, ATG5, and LC3B expression in AGS and AGS-HR cells. β-actin was used as a protein loading control. (**D**) EZH2 binding on HIF-1α promoter using chromatin immunoprecipitation (ChIP) with or without APG- or GSK-343 (10 μM, 24 h)-induced AGS-HR cells. (**E**) The interaction between HIF-1α and EZH2 using co-immunoprecipitation (co-IP) in APG-induced AGS-HR cells. (**F**–**H**) AGS and AGS-HR cells were transfected with EZH2 siRNA (30 nM, 24 h) and treated with APG (50 μM, 24 h). Cell viability and LDH activity were determined using WST-1 and LDH assays, respectively; * *p* < 0.05. EZH2, ATG5, and LC3B were detected using the Western blot assay. β-actin was used as a protein loading control. (**I**–**K**) AGS and AGS-HR cells were treated with APG (50 μΜ, 24 h) and/or GSK-343 (10 μM, 24 h). Cell viability and LDH release analyses were performed using WST-1 and LDH cytotoxicity assays; * *p* < 0.05. Western blot analysis was used to validate the targeted changes in EZH2, HIF-1α, ATG5, and LC3B expression in AGS and AGS-HR cells. β-actin was used as a protein loading control.

Hypoxia frequently mediates chemoresistance and tumor aggressiveness via the induction of HIF-1α in the tumor microenvironment, and targeting HIF-1α is a potential strategy to increase the effect of tumor chemotherapy [47]. To identify whether APG regulates the expression of HIF-1α under hypoxia in AGS cells, the WST-1 assay, LDH assay, and Western blot analyses were performed. Interestingly, APG treatment decreased cell viability and increased LDH release under normoxia and hypoxia to a greater extent than the control treatment (Figure 7A,B). In Western blot analyses, APG treatment decreased EZH2 and HIF-1α and increased ATG5 and LC3B under normoxia and hypoxia to a greater extent than the control treatment (Figure 7C). To identify the binding of EZH2 on the *HIF-1α* gene, we performed quantitative chromatin immunoprecipitation (qChIP) to identify EZH2 binding on the *HIF-1α* promoter in GC cells (Figure 7D). Chromatin samples from treatments with APG and the EZH2 inhibitor GSK-343 were immunoprecipitated with EZH2 antibody. The result indicates that EZH2 was able to bind the *HIF-1α* promoter under hypoxia exposure. However, APG and GSK-343 showed decreased binding of EZH2, and APG + GSK-343 treatment showed further reduced binding of EZH2 (Figure 7D). To identify the interaction of EZH2 and HIF-1α under normoxia and hypoxia exposure in APG-treated GC cells, co-immunoprecipitation (IP) using antibodies for EZH2 and HIF-1α was performed (Figure 7E). Our finding indicates that EZH2 and HIF-1α interact under hypoxia exposure in AGS cells; however, APG treatment inhibited the interaction between EZH2 and HIF-1α. To further confirm whether APG mediates autophagic cell death by inhibiting EZH2 under normoxia and hypoxia in AGS cells, the cells were transfected with EZH2-specific siRNA and then treated with APG. The cell viability assay, LDH assay, and Western blot analysis were performed. In EZH2 knockdown hypoxia-resistant AGS (AGS-HR) cells, APG decreased cell viability and enhanced LDH release to a greater extent than the control (Figure 7F,G). In Western blot analyses, APG increased ATG5 and LC3 lipidation in EZH2 knockdown AGS-HR cells to a greater extent than the control (Figure 7H). Our results suggest that APG mediates autophagic cell death via the activation of the PERK axis and inhibition of EZH2 and HIF-1α under normoxia and hypoxia in GC cells. To further confirm whether APG mediates the suppression of EZH2, a pharmacological experiment using GSL-343 (a selective inhibitor for EZH2) was performed. GSK-343 decreased cell viability and increased LDH release and LC3-II levels in APG-treated AGS-CTL and -HR cells. Western blot analyses indicated that GSK-343 plus APG decreased EZH2 and HIF-1α and increased ATG5 and LC3-II levels in AGS-CTL and -HR cells (Figure 7I–K). Therefore, APG induces autophagic cell death by inhibiting EZH2 and HIF-1α in AGS-CTL and -HR cells.

## 3. Discussion

An increasing number of reports indicate that the natural flavonoid APG is a powerful anti-cancer agent in various cancer types, such as pancreatic, colon, and gastric cancers [48,49,50]. However, the detailed molecular mechanisms underlying APG-mediated cell death still remain unclear and have not been studied. Our finding herein shows that APG was associated with ER stress- and autophagy-related cell death in GC cells, and to our knowledge, this is the first demonstration to investigate whether APG mediates autophagic cell death by regulating the PERK–eIF2α–ATF4–CHOP signaling in GC cells.

Many flavonoids have biological activities, such as cell death, angiogenesis, and cell proliferation, since cytotoxic anti-cancer effects of natural flavonoids have been noted in various cancer cell types [51]. Flavonoids have shown powerful effects in overcoming chemoresistance against chemotherapy and blocking cancer cell growth via various apoptosis pathways, such as the intrinsic apoptosis pathway, extrinsic apoptosis pathway, mitochondrial apoptosis pathway, ER stress, ROS, and autophagy [52].

The mechanistic target of rapamycin complex 1 (mTORC1), consisting of raptor, deptor, Pras40, and mLST8, relates to nutrients and growth factors, supports homeostasis, and is an important regulator of the autophagy process [53]. Under various stresses, inactivation of mTOR signaling causes the interaction between AMPK and ULK1 and promotes autophagy [54]. When we examined mTOR and AMPKα pathways in APG-treated AGS and SNU-638 cells, APG was found to induce the decrease in p-mTOR and p-P70S6K and the increase in p-AMPKα and ULK1. In the present study, APG mediated autophagic cell death, but autophagy knockdown against AMPKα, ULK1, ATG5, and LC3B suppressed cell death by increasing cell viability and reducing LDH release in APG-treated GC cells. Furthermore, APG treatment inhibited the changes in cell viability and LDH release, blocked the increased LC3B and ATG5, and reduced p62 expression after autophagy knockdown by LC3B- or ATG5-specific siRNAs.

Recent evidence suggests that flavonoids typically exert their anti-cancer activity via ER homeostasis–mediated ER stress [55]. Quercetin mediates ER stress-related cell death via eIF2α/CHOP signaling in ovarian and cervical cancer cells [56,57]. Kaempferol mediates caspase-3-dependent cell death and inhibits cell proliferation and survival via the GRP78/CHOP axis in hepatocarcinoma and breast cancer [12,58]. In the present study, APG was found to cause ER stress-related cell death by regulating PERK or CHOP signaling in GC, AGS, and SNU-638 cells. PERK or CHOP inhibition blocked the reduction in cell viability, increase in LDH release, and autophagy processes, such as LC3 lipidation in APG-treated AGS and SNU-638 cells. These results suggest that APG causes autophagic cell death via ER stress.

APG generates intracellular ROS release in colorectal cancer cells, and it causes various cell death types, including cell cycle arrest, chromatin condensation, MMP loss, intracellular Ca^2+^, annexin-v-positive cells, and ER stress-related cell death (CHOP and DR5) [59]. In estrogen receptor-positive MCF-7 cells and negative MDA-MB-231 cells, APG shows powerful anti-cancer properties, such as DNA damage and oxidative stress [60]. Overexpression of SPOCK1 in vitro and in vivo mediates chemoresistance by inducing the EMT phenotype; however, APG treatment dramatically blocked pancreatic cancer cell chemoresistance, migration, metastasis, tumor growth, proliferation, invasion, and cell survival [61]. With cisplatin-resistant colorectal cancer cells in vitro and in vivo, APG induces autophagic cell death, overcomes cisplatin resistance, and blocks xenograft tumor growth by inhibiting the mTOR pathway [62]. Many tumors have hypoxic conditions, and these are strongly associated with therapeutic resistance, tumor growth, metastasis, and immune dysfunction [63]. The hypoxic tumor microenvironment frequently mediates the up-regulation of hypoxia-inducible factors (HIFs), and hypoxia-mediated HIFs regulate diverse cellular signaling pathways, including oxygen homeostasis and vascularization [64]. Therefore, targeting HIF-1 has become an attractive therapeutic approach for the design and development of novel candidates for treating cancer [65]. Recent reports indicated that many dietary flavonoids inhibit HIF-1 expression by reducing side effects [66]. APG inhibited the expression of HIF-1α and mediated cell death through the suppression of STAT3 under hypoxia in breast cancer cells, suggesting that APG is a powerful anti-cancer drug to overcome hypoxia-induced chemoresistance [67,68]. In pancreatic cancer cells, APG suppresses the expression of HIF-1α and VEGF and induces cell death via the PI3K/Akt/GSK-3 signaling under both normoxia and hypoxia [36]. Our results show that APG mediated autophagic cell death by inhibiting EZH2 and HIF-1α under both normoxia and hypoxia in GC cells. Furthermore, EZH2 knockdown induced autophagic cell death via the inhibition of HIF-1α under both normoxia and hypoxia in APG-treated GC cells to a greater extent when compared with the control.

## 4. Materials and Methods

### 4.1. Materials

Apigenin (APG), Z-VAD-FMK, 3-methyladenine (3-MA), chloroquine (CQ), thapsigargin (TG), compound C, and GSK-343 were purchased from Sigma Chemical (St. Louis, MO, USA).

### 4.2. Cell Culture

The human GC cell lines (AGS, SNU-216, NCI-N87, SNU-638, MKN-7, and MKN-74) and lung normal cell line MRC5 were purchased from the Korean Cell Line Bank (Cancer Research Center, Seoul National University, Seoul, Korea). Cells were cultured in RPMI1640 medium (Welgene, Korea) supplemented with 10% fetal bovine serum (JR Scientific) and 100 μg/mL antibiotics (100 U/mL penicillin and 100 μg/mL streptomycin, Welgene) in a 5% CO_2_ humidified incubator at 37 °C.

### 4.3. Cell Viability Assay

The WST-1 assay was performed according to the manufacturer’s instructions (Roche, Mannheim, Germany) with 10 μL of WST-1 reagent added to each well of a 96-well plate (1 × 10^4^ cell/well). After 1 h of incubation using a CO_2_ incubator, the conversion of WST-1 reagent into chromogenic formazan was evaluated with a spectrophotometer (Molecular devices, USA). On day 1 after cell seeding, cells were treated with various doses of apigenin (Sigma) (30, 50, and 70 µM) at various time points (8, 16, and 24 h). Autophagy inhibitors 3-MA (Sigma, 5 mM), chloroquine (Sigma, 20 µM), compound C (Sigma, 2 µM), and SBI-0206965 (Sigma, 10 μM) were added sequentially to FBS-free medium for 24 h to inhibit autophagy. A pan-caspase inhibitor, Z-VAD-FMK (R&D Systems, 50 μM), was added to the FBS-free medium for 24 h to inhibit apoptosis. Cells were treated with an ER stress inducer, thapsigargin (Sigma, 3 μM, 24 h), along with the FBS-free medium to activate ER stress, and an Ezh2 inhibitor, GSK-343 (Sigma, 10 μM, 24 h), was added to activate autophagy via Ezh2 inhibition.

### 4.4. LDH Assay

AGS and SNU-638 cells (1 × 10^4^ cells/well) were seeded into a 96-well plate with growth medium. To determine the LDH (Thermo Scientific Pierce) activity in supernatants, 100 μL of reaction mixture was added and incubated for 30 min in a dark room. The LDH activity was measured by the absorbance of the samples at 490 nm or 492 nm using an ELISA reader.

### 4.5. Transfection

AGS and SNU-638 cells (3 × 10^5^ cell/well) were transfected with double-stranded siRNAs (30 nmol/mL) of LC3B, ATG5, ULK1, GRP78, PERK, EZH2 (Santacruz), and CHOP (Bioneer) in a 6-well plate for 24 h by the Lipofectamine 2000 (Invitrogen) method according to the manufacturer’s protocol and were then recovered in RPMI1640 medium (Welgene) containing 5% fetal bovine serum (Gibco) and 100 μg/mL antibiotics (100 U/mL penicillin and 100 μg/mL streptomycin, Gibco) for 24 h. After recovering, viable cells were calculated by the WST-1 assay.

### 4.6. Caspase-3 and -9 Activity Assay

AGS and SNU-638 cells (1 × 10^4^ cells/well) were seeded into a 96-well plate with growth medium. To determine the caspase-3 and -9 activity (Minneapolis, MN, USA, R&D Systems Inc.) after APG treatment, cell lysates (50 μg proteins) were incubated to check relative caspase activity using Caspase-3 and Caspase-9 Colorimetric Assay Kits (Minneapolis, R&D Systems Inc.) following the manufacturer’s instructions.

### 4.7. Isolation of Total RNA and Protein

Total RNA (approximately 50–100 mg) from GC cells (2 × 10^6^ cell/well) in a 100 mm cell culture dish was prepared using TRIzol according to the manufacturer’s protocols (Invitrogen, Carlsbad, CA, USA). Protein cell lysates were collected in RIPA buffer containing a protease inhibitor cocktail (Sigma) on ice for 30 min and were passed through an 18-gauge needle and spun down. The supernatant was analyzed for protein content using the BCA method (Thermo scientific, Pierce BCA Protein Assay Kit, Rockford, IL, USA).

### 4.8. Real-Time PCR and Western Analysis

LC3B, p62, ATF4, and CHOP expression level was measured by real-time PCR using cDNA synthesized from 5 μg of total RNA and a reverse transcription kit (Promega, Madison, WI). Reactions were performed in triplicate for each sample using ABI Power SYBR Green PCR Master Mix (Applied Biosystems) with LC3B-specific primers (5′-AGCAGCATCCAACCAAAATC-3′ (sense) and 5′-CTGTGTCCGTTCACCAACAG-3′ (antisense)), p62-specific primers (5′-GTGAATTCGCTCGCCGCTCGCTAT-3′ (sense) and 5′-CGTCTCGAGTGCCTGCTGACAACACCTA-3′ (antisense)), ATF4-specific primers (5′-AAGCCTAGGTCTCTTAGATG-3′ (sense) and 5′-TTCCAGGTCATCTATACCCA-3′ (antisense)), and CHOP-specific primers (5′-ATGAGGACCTGCAAGAGGTCC-3′ (sense) and 5′-TCCTCCTCAGTCAGCCAAGC-3′ (antisense)) on a Roche LightCycler 96 (Roche). RNA quantity was normalized to β-actin primers (5′-AAGGCCAACCGCGAGAAGAT-3′ (sense) and 5′-TGATGACCTGGCCGTCAGG-3′ (antisense)). Gene expression was quantified according to the 2^−ΔΔCt^ method. To conduct the Western blot assay, GC cell lines were solubilized in the radioimmunoprecipitation assay (RIPA) lysis buffer (50 mM/L Tris-HCl (pH 7.4), 150 mM/L NaCl, 1% NP40, 0.25% sodium deoxycholate, 1-mM/L phenylmethylsulfonylfluoride (PMSF), 1 mM/L sodium orthovanadate, and 1× sigma protease inhibitor cocktail), and the protein content was estimated using a standard bicinchoninic acid assay. Equal amounts of protein (20 μg) were size fractionated by 8–15% SDS-PAGE and then transferred onto an NC membrane (Millipore Corporation, Billerica, MA, USA). Membranes were blocked by incubation for 30 min with 5% skim milk/PBS-T (PBS with 5% powdered milk (BD) and 1% Tween20 (Sigma)) and incubated overnight at 4 °C with primary antibodies diluted in 1× PBST buffer. The following primary antibodies were used: β-actin, Bcl-2, Beclin-1, ULK1, eIF2α, GRP78, ATG5 (Santa Cruz, 1:1000); LC3B (Sigma, 1:1000); CD63 (Abcam, 1:1000); EZH2, HIF-1α, cleaved caspase-3, cleaved caspase-9, ATF4, p62, p-AMPKα (Thr172), AMPKα, p-mTOR (Ser2448), mTOR, p-ULK1 (Ser555), PERK, p-PERK (Thr980), p-eIF2α (Ser51), and CHOP (Cell Signaling, 1:1000). The membranes were washed three times with PBST buffer. A secondary antibody diluted in PBST or TBST buffer was added, and incubation was conducted for 40 min at room temperature. The following secondary antibodies were used: anti-rabbit IgG HRP-linked antibody and anti-mouse IgG HRP-linked antibody (KPL, 1:6000). The membranes were washed six times with PBST buffer for 1 h. The blots were visualized using a Western chemiluminescent HRP substrate (Millipore).

### 4.9. Cyto-ID Staining

AGS and SNU-638 cells (2 × 10^5^ cells/well) in a 6-well plate were seeded and treated with 50 µM APG for 8 h. Additionally, cells were stained with Cyto-ID autophagy detection kit (Enzo Life Science). An autophagosome-positive puncta was observed by confocal microscopy. Confocal microscopy was performed using a ZEISS LSM5 PASCAL confocal microscope with 405 and 488 nm excitation lasers.

### 4.10. Immunoprecipitation (IP) Assay

We extracted cell lysates from AGS and SNU-638 cells (2 × 10^6^/well) on a 100 mm cell culture plate in an IP buffer (pH 7.5) containing 50 mM Tris-HCl, 250 mM NaCl, 5 mM EDTA, 0.5% (*v*/*v*) NP-40, and protease inhibitor cocktail (Sigma). We incubated the antibodies anti-Bcl-2 (Santa Cruz), anti-BECN-1 (Santa Cruz), HIF-1α, and EZH2 (Cellsignaling) with lysate at 4 °C for 16 h. We used protein A/G Plus agarose (Santa Cruz) to pull down immunocomplexes. We washed precipitates three times with IP buffer. We resolved the immunoprecipitated proteins using 12% SDS-PAGE and analyzed them.

### 4.11. Chromatin Immunoprecipitation (ChIP) Assay

ChIP assays were performed using EZ ChIP Chromatin Immunoprecipitation Kit (Millipore, Billerica, MA, USA) as described in the supplier’s protocol. Briefly, the cross-linked chromatin was sonicated after cell lysis and then incubated overnight at 4 °C with antibodies against EZH2 (Cellsignaling). The immunocomplex was precipitated with protein A agarose (Millipore), and the beads were washed, sequentially treated with 10 µL of RNase A (37 °C for 30 min) and 75 µL of proteinase K (45 °C for 4 h), and incubated at 65 °C overnight to reverse cross-link the chromatin. The DNA was recovered by phenol–chloroform extraction and co-precipitation with glycogen and was then dissolved in 50 µL of Tris-EDTA (TE) buffer. PCR primers (5′-CAGCCGCTGGAGACACAAT-3′ (sense) and 5′-GGTACTTCCTCAAGTTGCTGGT-3′ (antisense)) were designed to amplify the EZH2 binding site at the *HIF-1α* gene promoter. Quantitative PCR conditions were 40 cycles at 94 °C for 40 s, 60 °C for 1 min, and 72 °C for 40 s.

### 4.12. Measurements of Intracellular Calcium Release

AGS and SNU-638 cells (1 × 10^4^ cells/well) were seeded into a 96-well plate with growth medium and treated with APG in a time- and dose-dependent manner. To determine the intracellular calcium release (Calcium Assay Kit (Colorimetric); Abcam), chromogenic reagent and calcium assay buffer were added and incubated for 10 min at room temperature. The intracellular Ca^2+^ release was measured by the absorbance of the samples at 575 nm using an ELISA reader (Molecular Devices).

### 4.13. Exosome Isolation

The cells were treated with APG at the dose shown (30 and 50 µM), and exosomes were obtained from the supernatant of APG-treated AGS and SNU-638 cells according to the manufacturer’s protocol (Total Exosome Isolation Reagent (for cell culture media), Thermo Fisher Scientific). Protein concentration was measured using the BCA method (Thermo Scientific). The protein loading samples (10 μg) were also quantified by Ponceau S staining and were subjected to Western blotting. Positive exosomes were identified using the exosome marker CD63.

### 4.14. Normoxia and Hypoxia Exposure

The cells plated on a culture dish were placed into a modulator incubator chamber (Billups rothenb, MIC-101) sealed with rubber stoppers. For hypoxia induction, cells were cultured in hypoxia chambers (Sanyo; containing 1% O_2_, 5% CO_2_, 94% N_2_). For the normoxia condition, cells were cultured in an incubator containing 5% CO_2_ and ~20% O_2_.

### 4.15. Animals

For the animal study, five-week-old female athymic BALB/c nude mice (*nu/nu*) were purchased from OrientBio, Inc. (Daejeon, Korea) and maintained for 1 week with free access to sterile standard mouse chow (NIH-7 open formula) and water before use. Mice were housed randomly at 50% ± 20% humidity and approximately 21 °C ± 2 °C on a 12 h light–dark cycle (*n* = 5 mice/group). All animal experimental procedures were performed according to the National Institutes of Health guidelines and a protocol approved by the Institutional Animal Care and Use Committee of Kyung Hee University.

### 4.16. Tumor Xenograft Mouse Models

For the mouse xenograft experiment, six-week-old mice were inoculated with the AGS human gastric cancer cell line by subcutaneously (sc) implanting 1 × 10^7^ cultured cells into the right thigh. Six days later, mice were grouped randomly (*n* = 10 per group) and APG (200 or 300 mg/kg) was administered intraperitoneally (ip) once a day for two days. Tumor sizes on two axes (L, longest axis; W, shortest axis) were measured three times per week using vernier calipers. Tumor volume was calculated using the formula (L × W^2^)/2 (mm^3^).

### 4.17. Statistical Analysis

All results were confirmed in at least three independent experiments. One-way analysis of variance (ANOVA) with Tukey’s post hoc test was used for between-group comparisons of the means of quantitative data, and *p* < 0.05 was considered statistically significant.

## Data Availability

Not applicable.
