# Correction: Kim, T.W.; Lee, H.G. Apigenin Induces Autophagy and Cell Death by Targeting EZH2 under Hypoxia Conditions in Gastric Cancer Cells. *Int. J. Mol. Sci.* 2021, *22*, 13455

**DOI:** 10.3390/ijms27062686

**Published:** 2026-03-16

**Authors:** Tae Woo Kim, Hee Gu Lee

**Affiliations:** 1 Immunotherapy Research Center, Korea Research Institute of Bioscience and Biotechnology, Daejeon 34141, Republic of Korea; 2 Department of Preventive Medicine, College of Korean Medicine, Kyung Hee University, 1 Hoegi, Seoul 130-701, Republic of Korea; 3 Department of Biomedicine & Health Sciences, College of Medicine, The Catholic University of Korea, Seoul 06591, Republic of Korea; 4 Department of Biomolecular Science, University of Science and Technology, Daejeon 34113, Republic of Korea

In the original publication [1], there was a mistake in Figure 3F as published. Unintentionally, the blot images of β-actin were incorrectly selected in the original manuscript. The corrected Figure 3F appears below. The authors state that the scientific conclusions are unaffected. This correction was approved by the Academic Editor. The original publication has also been updated.

## Figures and Tables

**Figure 3:**
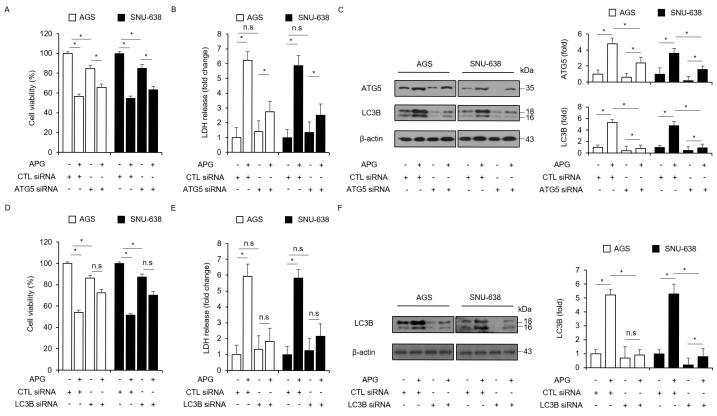
Autophagy inhibition regulates APG-induced cell death. (**A**–**F**) After AGS and SNU-638 cells were transfected with LC3B and ATG5 siRNAs, cell viability, LDH production, and Western blot analyses were performed with/without APG (50 μM, 24 h) treatment. Cell viability and LDH activity were determined using WST-1 and LDH assays, respectively; * *p* < 0.05, n.s; no significant. Western blotting was performed to identify the autophagy-related genes ATG5 and LC3B in APG-treated ATG5 or LC3B knockdown cells. β-actin was used as a protein loading control.
